# Treating childhood intermittent distance exotropia: a qualitative study of decision making

**DOI:** 10.1186/s12886-015-0087-y

**Published:** 2015-08-22

**Authors:** Jan Lecouturier, Michael P. Clarke, Gail Errington, Nina Hallowell, Madeleine J. Murtagh, Richard Thomson

**Affiliations:** Institute of Health and Society, Newcastle University, Baddiley-Clark Building, Richardson Road, Newcastle upon Tyne, NE2 4AX UK; Newcastle Eye Centre, Royal Victoria Infirmary, Newcastle upon Tyne, NE1 4LP UK; Faculty of Medicine & Health Sciences, University of Nottingham, Queen’s Medical Centre, Nottingham, NG7 2UH UK; Centre for Population Health Sciences, Medical School, The University of Edinburgh, Teviot Place, Edinburgh, EH8 9AG UK; School of Social and Community Medicine, University of Bristol, Oakfield House, Oakfield Grove, Clifton, Bristol, BS8 2BN UK

**Keywords:** Shared decision making, Intermittent distance exotropia, Children, Qualitative research, Treatment uncertainty

## Abstract

**Background:**

Engaging patients (parents/families) in treatment decisions is increasingly recognised as important and beneficial. Yet where the evidence base for treatment options is limited, as with intermittent distance exotropia (X(T)), this presents a challenge for families and clinicians. The purpose of this study was to explore how decisions are made in the management and treatment of X(T) and what can be done to support decision-making for clinicians, parents and children.

**Methods:**

This was a qualitative study using face to face interviews with consultant ophthalmologists and orthoptists, and parents of children with X(T). Interview data were analysed using the constant comparative method.

**Results:**

The drivers for clinicians in treatment decision-making for X(T) were the proportion of time the strabismus is manifest and parents’ views. For parents, decisions were influenced by: fear of bullying and, to a lesser degree, concerns around the impact of the strabismus on their child’s vision. Uncertainty around the effectiveness of treatment options caused difficulties for some clinicians when communicating with parents. Parental understanding of the nature of X(T) and rationale for treatment often differed from that of the clinicians, and this affected their involvement in decision-making. Though there were good examples of shared decision-making and parent and child engagement some parents said the process felt rushed and they felt excluded. Parents reported that clinicians provided sufficient information in consultations but they had difficulties in retaining verbal information to convey to other family members.

**Conclusions:**

Overall parents were happy with the care their child received but there is scope for better parent and (where appropriate) child engagement in decision-making. There was an expressed need for written information about X(T) to reinforce what was given verbally in consultations and to share with other family members. Access could be via the hospital website, along with videos or blogs from parents and children who have undergone the various management options. A method of assisting clinicians to explain the treatment options, together with the uncertainties, in a clear and concise way could be of particular benefit to orthoptists who have the most regular contact with parents and children, and are more likely to suggest conservative treatments such as occlusion and minus lenses.

**Electronic supplementary material:**

The online version of this article (doi:10.1186/s12886-015-0087-y) contains supplementary material, which is available to authorized users.

## Background

Intermittent distance exotropia (X(T)) is a form of childhood squint (strabismus) with an estimated prevalence of 1 % in children under 11 years [1]. The strabismus is exacerbated when the child focuses on distant objects, is tired or daydreaming, and may be accompanied by monocular eyelid closure in bright sunlight. The natural history of X(T) is uncertain; study findings vary and most are methodologically flawed [2]. A current large scale randomised controlled trial of occlusion for the treatment of X(T) in the United States, due for completion in 2015, may answer this question, as a secondary objective is to determine the natural history of X(T) in patients aged 3 to 11 years [3].

The longer term risks to a young child with X(T) are amblyopia (decreased vision due to developmental anomaly resulting from the strabismus) or loss of binocular vision. For this reason a child’s vision and strabismus will be regularly monitored. Psychosocially, a key concern is childhood bullying resulting from stigma associated with the appearance of the strabismus. However, research has tended to focus on the impact of manifest strabismus [4–7]. Due to its intermittent nature it is not certain if stigma and bullying are an issue for the majority of children with X(T).

Children with X(T) normally present between the ages of 2 to 4 years when divergent misalignment or monocular eye closure is observed [8]. In the UK, options for management of X(T) include observation, orthoptic exercises to strengthen binocular single vision, occlusion, minus lenses, prisms and surgery. Evidence for superiority of any of these options is sparse. A literature review [9] concluded that conservative management options were a viable alternative or adjunct to surgery, but that further research was needed to determine the “dosage” of occlusion and the effectiveness of minus lenses. A recent study [10] that compared part-time occlusion with observation in children aged 3–10 years of age reported a slightly lower rate of deterioration with the latter; they concluded that X(T) could be reasonably managed with either option. A Cochrane review of interventions for X(T) [2] identified only one randomised controlled trial in which unilateral and bilateral surgery were compared; the former was found to be more effective in correcting the strabismus [11]. Due to the lack of robust evidence, the authors conclude that issues around the optimum age for surgery remain unresolved and the effectiveness of non-surgical interventions remains unclear. A recent systematic review concluded that, given the limited evidence base, better designed studies are required to address the question of the most effective management and treatment of childhood X(T) and that consensus is required on what constitutes a successful outcome [12].

This uncertainty around the most appropriate management of X(T) has implications for families and clinicians making decisions about treatment. Shared decision making (SDM), engaging patients (parents/families) in decisions about treatment when there are alternative options - particularly where the risks, benefits and consequences of the options vary - is increasingly recognised as important and beneficial [13] and is embedded in health policy [14, 15]. SDM may be differentiated from more traditional models of decision making as it incorporates patient preferences and values into treatment decisions [13]. In the treatment of X(T), where the evidence base for alternative options is limited, SDM presents a challenge.

Considering the uncertainty surrounding the management options, we interviewed parents and clinicians (orthoptists and consultant ophthalmic surgeons) to gain an understanding of how treatment decisions about X(T) are currently made. We also explored the factors and issues that shape these decisions to illuminate how to better support those involved.

## Methods

This was a qualitative study using one-to-one in-depth interviews (see Additional file 1). A purposive sample was used to ensure we included the views of the parents whose children were being managed across a range of options: monitoring, occlusion, minus lenses and surgery.

Topic guides – a broad outline of key areas for discussion - for the clinician and parent interviews were developed with the input of a study advisory group, which included parent and child representatives, clinicians independent of the project and a hospital trust governor. The overarching themes explored in the interviews were impact of X(T), experience and understanding of treatment, and decision making about X(T). Additionally, clinicians were asked to describe the organisation and delivery of care, and parents how the X(T) was identified and their route into the hospital system. Both parents and clinicians were asked to record their own preferences for engagement in treatment decisions using the Control Preference Scale [16] – which was designed to assess preferences for involvement in decision making.

Consent was obtained prior to the interviews. Interviews were digitally audio-recorded with the permission of the interviewee and transcribed verbatim. NVivo (a computer software package) was used as a data management tool to assist with coding the interviews [17]. Data were analysed using a constant comparative method [18], a process of comparing and contrasting themes elicited from the data either across or within interviews. The constant comparative method also allowed us to assess when data saturation (i.e. when no new information emerges) was achieved: at the point when data saturation was reached we ceased recruitment. Ethical approval was granted by NHS Research Ethics Committee – Sunderland.

### Recruitment

#### Clinicians

Consultant ophthalmic surgeons and orthoptists responsible for the care of children with X(T) were identified and approached by the clinical leads and chief orthoptists in four ophthalmology centres in the North of England. They were then contacted by a member of the study team to ask if they would be happy to participate and be interviewed.

#### Parents

Orthoptists in two of the four ophthalmology centres identified children up to the age of 12 years diagnosed with X(T) in the out-patient clinics and approached parents about the study. Parents interested in learning more about the study were given a brief study information sheet and asked if they could be contacted by a member of the research team. Parents who agreed were sent full study information and telephoned a week later, to answer any questions about the study and to ascertain if they wished to participate.

Four months into data collection we found many children identified were not receiving any active treatment for their strabismus and were being observed over time– this was especially so for the under 4’s - and data saturation was reached for this group. Consequently, for the remainder of the data collection period, we focused upon children aged between 4 and 12 years who were having active management or treatment of their strabismus, i.e. minus lenses, occlusion or surgery. We reached data saturation for the surgical group but there were very few children attending clinic over the recruitment period being managed with occlusion or minus lenses (Table 1). The data from interviews with parents are presented according to treatment groups.

**Table 1:** Treatment of strabismus by age group

	Monitored	Occlusion	Minus lenses	Pre/post surgery
Under 4 years	10	1	0	0
4-12 years	6	0	6	4/10

## Results

Thirteen orthoptists, eight ophthalmic surgeons and 37 parents (11 parents of under 4’s and 26 of children aged 4–12 years) were interviewed. Table 1 indicates the children’s treatment by age group.

### Referral pathway in the UK

There are two main referral routes to the eye clinic: family doctor (usually where the strabismus is noticed by the parent or health visitor) and school screening clinics conducted in most areas by orthoptists. Children are screened in School Reception Year (around the age of 4 years) for visual abnormalities. Parents receive a letter informing them of the outcome of the screening and whether they need to take any further action.

In two of the four sites every child with an X(T) sees the consultant ophthalmic surgeon early in the care pathway. In the other two sites this is determined by the orthoptists and depends upon the features of the strabismus or whether the parents specifically ask to speak to the consultant. Often the children are managed by the orthoptists and the consultant is involved only at the beginning and/or when surgery is indicated.

### Making treatment decisions

In three of the four sites, the decisions about which conservative treatments to offer, such as observation, occlusion and minus lenses, are the preserve of the orthoptists. However, when surgery is considered consultants are always involved in discussions with parents and children.

#### Clinicians’ perspectives

When asked to record their own preferences for engagement in treatment decisions the majority (12 of 21) expressed a preference for sharing the decision with parent/child (Table 2) but this was not always borne out in the interview data. Shared decision making has been defined as ‘an approach where clinicians and patients share the best available evidence when faced with the task of making decisions, and where patients are supported to consider options, to achieve informed preferences‘ [13]. Yet in some cases the decisions appeared to be driven by the clinician and in others were more in line with informed (i.e. where the parents were given information and asked to make the decision themselves) rather than shared decision making:

**Table 2:** Orthoptist and ophthalmic surgeons’ preferences for decision making – Control Preference Scale

	Orthoptists	Ophthalmic surgeons
Patient/parent to make the final decision	2	
Patient/parent to make the final decision after seriously considering my opinion	4	3
I share responsibility with patient/parent for deciding	7	4
I make the final decision but seriously consider the patient’s/parent’s opinion		
I make all decisions		1

*‘I think you sort of guide them and that’s why it’s very important that you are thorough and then they will listen to your advice and expert advice. So in some ways we guide them into where we think is the best way to go.’****Orthoptist***

*‘I just say “As your consultant I can tell you what the pros and cons are, this is what normally is suggested, this is what we do to children, whether you should have it done for your child, it’s your choice.”’****Consultant***

There was variation across and within centres in the extent and quality of parental engagement in treatment decisions. For example, in some cases there was a sense of a one-way process where clinicians conducted numerous assessments and conveyed much information to parents but with minimal interaction with regard to decision-making. This could be due to a lack of awareness: some clinicians appeared to consider the parental element of the Newcastle Control Score [19] - where parents are asked how often they notice the X(T) – as a form of parent engagement. Albeit a minority view, one clinician expressed frustration at parents who ‘go round and round’ and cannot make a decision, especially when this takes up extra clinic time. Rather than explore why it was difficult to make this decision, the response was to suggest they discuss it within the family and, if they want to proceed, telephone to book a date for surgery.

Some clinicians reinforced the importance of continuity of care and how familiarity can engender parent engagement in treatment decisions.

*‘… having the same continuous person to see is actually better than having a different person each time just going through a myriad of tests. And I think if one person saw them you probably would miss out some of the tests and spend more time talking, I think that’s more beneficial.’****Orthoptist***

There were a few examples of clinicians eliciting parent and child preferences for treatment and discussing what would work best for the child and family.

*‘I would ask them about the child “What do you think your child would comply with best?” because they know the child better. “What do you think is more suitable for your home environment?” because of the other commitments parents have and how easy it is for them to do six hours of occlusion if they work full time and their child is not with them. So sort of try and look into their own environment and which of these three [treatments] are best.’****Orthoptist***

With regard to children, the majority view was that, in this condition, most are too young to participate in the consultation and decisions. Although orthoptists try to include children, they need to determine how much the child understands about what is happening and that can be difficult when they are unaware there is a problem with their eye. In addition, a policy of always involving or informing the child is not always sought by parents, as some do not want their child to know they will be having surgery.

#### Factors influencing treatment decision-making

Clinicians talked about a number of clinical and non-clinical factors that influenced their treatment decisions.

Clinical features, such as deterioration in vision, angle of the strabismus and how well the child is controlling the strabismus, influenced the choice of treatment. However, obtaining accurate measures in young children was problematic. This, along with the variability in these measures between visits, led to children being observed for longer periods until the clinicians felt they had a true picture of the condition. As one orthoptist said ‘*you want a clinical decision to be made about surgery or treatment options based on the facts*’.

Uncertainty around the benefits of, and lack of confidence in, certain management options had an impact on what was offered to parents and children, although this varied within and between centres. There was also variation in the options available for the management of X(T). In some centres, the choices were monitoring or surgery, and other options were rarely used.

Some orthoptists and ophthalmic surgeons would more readily try conservative management, often driven by direct experience of success with a particular option. When options fail there was concern about the impact on parents and children. There was uncertainty about the benefits of conservative treatments for some and a sense of frustration because of the lack of robust evidence of effectiveness.

*‘What I don’t know and what I can’t tell parents is ‘This works in so many percentage of cases’ or ‘This works best in children with this type of problem’.’****Orthoptist***

This did not appear to be an issue for consultants; one consultant said that he is always honest with parents about the uncertainty around the optimum age to operate and felt they were happy with this explanation.

As might be expected, consultants had a greater preference for surgery, justified by the lack of evidence of any long-term benefit of conservative management. For some clinicians the decision to propose certain conservative management options was driven by a need to ‘buy time’ and delay surgery.

*‘Well I think that minus lenses and patching are probably only temporising measures and they’re probably not achieving a great deal apart from, em you know, giving the parents something to do while it gets better or not on its own.’****Consultant***

*‘In my experience we could do this short term until we could make a better decision on long term management which could be surgery, and if the child is too young then measurements may not be as accurate at that time to make that decision. … So from my point of view it’s buying us time rather than treating.’****Orthoptist***

Parents’ opinion was stated as a factor influencing decisions on management of X(T). More specifically, the extent to which the appearance of the strabismus is of concern to parents featured quite heavily in how both orthoptists and ophthalmologists managed a child’s strabismus.

*‘I mean you might decide at the outset just to watch someone because the parents don’t particularly fancy glasses and they’re not keen on an operation. And a child, who looks exactly the same, has exactly the same control and the same measurements might have glasses. And that’s basically after you’ve had a discussion with the parents about what they want to do.’****Consultant***

Although most consultants felt they had to consider the parents’ opinions, they reported that they would not conduct surgery unless there were clear clinical features that indicated this was the best option.

#### Support for parents in decision-making

Clinicians reported that on the first visit parents are seeking information on the aetiology of the strabismus and options for treatment. Anxieties diminished once parents were reassured, when they knew the diagnosis and that the condition would be monitored.

All believed that they understood parents’ concerns and these relate primarily to the appearance of the strabismus and the psychosocial consequences i.e. the potential for the child to be bullied because of their appearance. Secondary to this were worries about the impact of the strabismus on their child’s vision. The widespread view was that the vast majority of children are unaware they have a strabismus and, apart from a small number who are symptomatic (extra sensitivity to bright sunlight, double vision), most are not troubled by it. Few were convinced that the strabismus had any impact on a child’s educational development. The majority of clinicians thought that, in many cases, the younger children are less bothered by the X(T) than their parents.

*‘I think most of the kids are under seven and I don’t think they themselves are particularly fussed about it and I don’t think their peers at school particularly notice it. I think their parents are worried that it will be a source of bullying and difficulty making friends and that sort of thing.’****Consultant***

Clinicians believed parents received sufficient information about the treatment options; though some orthoptists said they often had to reiterate the information at each visit and certain parents needed to receive this piecemeal ‘*building on it gradually … trying to make sure they understand one concept before we introduce another*’. A number of clinicians used the clinical data to demonstrate change over time and the need to consider intervening.

*What I do personally, because I can relate to that as a parent, is to show them the prism bar and the measurements. …. So they can see how on a scale it changes, to help them understand that this is quite a lot for a child to control …. So bring things together from the previous visits and lead up to the option of surgery and “How do you feel about that?” and “You don’t have to make a decision now or even if you see the consultant it’s just a discussion and to see what else is available to you and your child”.’****Orthoptist***

In some sites orthoptists mentioned surgery to every new patient with X(T), to prepare parents and children should surgery be advised in the future. In others, they would only do so if the condition was severe.

Unless the X(T) was particularly severe, clinicians advised parents that *not* intervening posed no risk as long as the child was observed. There were no risks identified with the conservative treatments such as minus lenses or occlusion and, because of the uncertainty around success rates, clinicians did not expound the benefits. Explanation of the risks and benefits was more likely to be given in relation to surgery. In some sites the orthoptists gave a more in-depth explanation, in others the ‘bare bones’. In all sites the consultants met with parent and child to go over the information and answer any questions about surgery. Over and under-correction was a risk mentioned but some clinicians provided more information than others:

*‘I explain to them what surgery involves, that a lot of muscles around the eye, adjusting the muscles and that they might operate on both eyes not just one eye, they work as a pair. The risks of overcorrection - what would happen if they were overcorrected – double vision.’****Orthoptist***

*‘So I tend not to say ‘I can give a child double vision, which could be permanent, or amblyopia’, because it almost never happens. But I do mention overcorrection, I say it’s possible.’****Consultant***

Most appreciated the difficulties parents face, especially in decisions around surgery, and said they were willing to spend time answering their questions and took pains to emphasise that a decision is not critical at that point. A few of the orthoptists commented on the lack of continuity of care, with children and parents seeing someone different at each visit, and the negative impact this could have on decision making.

#### Parents’ perspectives

In almost half of the parents interviewed, their children were being actively observed (one of the conservative treatment approaches) by the ophthalmology centre staff. The parents were happy with this and did not consider it as an option that required a decision on their part. The ophthalmological centres responsible for the care of their children use the Newcastle Control Score [19] to monitor the strabismus. This has a home component where parents observe how often they notice the strabismus. Because of this most parents were aware that if the strabismus is present for 50 % of the day this is a trigger for more active management.

Parents also completed the control preference scale (Table 3). The majority (17/36) expressed a preference for making the decision themselves taking account of the clinicians’ opinions; 12 to share the decision with the clinician and a minority (6/36) that the clinician make the decision. The pattern was similar when looking only at families where the child had undergone, or was waiting to have, surgery. From the interviews, some parents considered the role of the clinicians was to provide information, advice and recommendations on management, others reflected more of a shared process where the decision was made ‘with the doctors’.

**Table 3:** Parents’ preference for decision making - Control Preference Scale

I make the final selection about which treatment my child receives.	1
I make the final selection of my child’s treatment after seriously considering the doctor’s opinion	17
The doctor and I share responsibility for deciding which treatment is best for my child	12
That the doctor makes the final decision about which treatment for my child, but seriously considers my opinion	5
Leave all decisions regarding my child’s treatment to the doctor	1

One parent could not decide between ‘shared responsibility’ and ‘doctor makes decision but considers parents’ opinion’ – this is excluded from the table

Other family members, such as grandparents, were mentioned as playing a role in decision making about the children’s treatment. In a few cases, parents said they had disagreed with each other about whether their child underwent surgery, though they had not openly disagreed with the surgeon; this was clearly a difficult situation that was ultimately resolved between the parents, apart from one family in which the child was old enough to make the final decision.

There was a high level of trust in the consultants and most parents were happy to take their advice, particularly when it came to the option of surgery.

*‘… the hospital was suggesting that was the route to go down. He did say that it was better to do it now. And I thought well he is the expert. ‘****4****–****12 group - surgery***

### Factors influencing treatment decision-making

In some cases parents were unaware that their child had a strabismus until they received a letter from the school screening clinic. Even within families, sometimes one parent could not see their child’s strabismus. A small number of parents felt the strabismus was of very little trouble to themselves or the child and were happy for their child to be monitored by the staff in the eye clinic. However, they did appreciate that the situation could change and they would then have to make a decision on treatment.

*‘Given how intermittent it is, it’s not a major issue for her. … But I think if we were in a situation where her eye was out more we would, I think it would obviously become much more of an issue for us, … I think our difficulty would be if we were doing something purely for cosmetic reasons and not medical reasons then I think we would find that an awkward decision to make.’****4****–****12 group - active monitoring***

However, this group were in the minority and most described a number of factors that had influenced treatment decision-making.

The majority of parents of pre- and post- surgery children gave fear of bullying about the strabismus when the child is older as the major influence on the decision to have surgery.

*‘It’s the bullying, because kids are cruel and they’re getting worse. I work with children and I thought well I don’t want [child] getting called names when she’s bigger because of her eyes.’****4****–****12 group - surgery***

Despite this fear, there were very few actual instances of bullying reported. The general opinion was that ‘kids are cruel’ and parents appeared certain that their child would be bullied in the future because they looked different. In many cases, there was a family history of strabismus but, apart from one parent, no one said that they or their family member had been subject to bullying or had been stigmatised because of the strabismus. A few mentioned adults they knew with a noticeable strabismus and that they did not want their child to reach adulthood and still have the strabismus.

The other main influence on decision-making was concern about their child’s vision. Some parents struggled with the notion of putting their child through a surgical procedure for reasons other than to improve or retain vision. They preferred conservative management, and their children were compliant and happy with the treatment.

*‘I’m of the view that if it was just for cosmetic reasons I don’t really see the need for her to have surgery. … (Child) is quite able to stand up for herself and I wouldn’t worry about anybody saying things to her or anything like that so I don’t think I would want surgery for just those reasons. If she needed the surgery to actually correct her vision then it would be something we’d have to consider.’****Under 4 group - occlusion***

Other factors were described as influencing treatment decisions. First, choosing an option that would minimise the ongoing impact on the child and family. For some, surgery was the preferred option as it offered a means of sorting out the problem in a single procedure – getting everything over and done with while the child is still young - negating the need to try different conservative options.

*‘It was better for her that way rather than trying different things, so really. . I mean she did want to have a try of glasses and I thought, her [sister] has glasses and she doesn’t like them at all and I’m not going to put her through glasses when she doesn’t really need to. … It’s out of the way and over and done with rather than prolonging the thing … she needed it done. And she’s young enough to forget about it when she’s older.****4****–****12 group - surgery***

Of course this was not always the case; there were parents where the procedure had not been successful after the first attempt and those whose child had undergone a second surgical procedure for that reason.

Risks of the anaesthetic, as well as the possibility of the strabismus being worsened as a result of surgery, were an issue for the majority of parents. Some parents thought that the surgery might damage the child’s eyesight and, if subsequent surgical procedures were required, this would have a further detrimental impact. Another issue was that more than one operation might be required with no guarantee of success.

*‘[They] did strongly advise us to have the operation … I was more surprised because [they] said that even if she had the operation sometimes doesn’t work … sometimes it can take two or three times … we just said we just don’t want to put her through all of that if it’s not even guaranteed.’****4****–****12 group – minus lenses***

In a few cases, other family members had undergone successful surgical intervention for strabismus. This appeared to raise expectations that this would also be the case for their own child and this experience influenced their decision-making.

Professional opinion was a key factor and some parents were influenced by the clinician’s view that it was best for the child to have the operation when they were younger. The reasons given were that: younger children recover better, are less likely to be emotionally traumatised by the experience and would forget about it over time. A few parents said the issue of potential bullying or clinicians mentioned teasing as a reason for considering surgery. Some parents had an understanding that there was a point where it would be too late to undergo surgery or the chance of success would be diminished as time went on.

### Support for decision making from clinicians

Clear information about the condition and the management options is a key component in good decision-making. When their child attends as a new patient parents are asked if they would like a copy of the letter sent to the family doctor. These outline their attendance, diagnosis and current treatment and do not include any details of discussion. None of the parents interviewed mentioned this as a source of information.

The majority reported that they were well informed by the staff in clinic, but relaying all of the information to a parent or family member not able to attend the clinics was difficult. A number of parents believed that a ‘lazy eye’ and strabismus were the same. Aware of other children successfully treated with occlusion for lazy eye, they were unclear as to why their own child’s strabismus could not be cured in the same way. None of these parents had asked about this when in clinic; some were concerned about asking what might be considered silly questions. Some parents had tried to find specific information via the internet but had uncovered information on a range of different types of strabismuses.

Some parents were unclear about the different treatment options and each possible management option was not always discussed. Surgery was mentioned as an option more often for the children who presented when they were over 4 years old, usually early in the care pathway and for some at the first visit. When surgery was discussed with parents of under-4’s, clinicians sometimes referred to it as a last resort or as an option only when reliable measurements could be obtained. In some cases surgery was the only treatment offered; others recalled occlusion or glasses being mentioned, but that neither were an option for their child (the reason was not always given but in some cases it was because the X(T) was too severe) and surgery was recommended. One parent was led to believe that surgery was for cosmetic reasons and, as she felt it was unnecessary in her child’s case, had resisted for a number of years. She was shocked when she last attended to hear differently:

*‘At his last appointment I was told it wasn’t for cosmetic reasons, surgery would be the only answer to actually correct it and that his eyes weren’t working together and surgery would be the only way that would help them work together. If I’d been told when we first went when he was four that his eyes would never work together I would have had it done then, but was led to believe it was more cosmetic to make it look better.’****4****–****12 group - surgery***

Although, from the clinicians’ accounts, a decision to proceed with surgery is rarely urgent, a few parents felt rather rushed in making a decision. In addition, some parents seemed to be floundering in the face of making a treatment decision for their child and did not feel they had the support of clinicians:

*‘I got the impression they wouldn’t do the surgery unless obviously we were happy about it. But it kind of felt like a bit of a responsibility, again, on us, to make that decision. I don’t know (laughs), I don’t really know what’s best. I can only think what I would feel if I was (child) I suppose, try and put yourself in her kind of position. When she’s older you wouldn’t want her to have her vision affected or have the eye looking, you know if it was right out.’****Under 4 – Minus lenses***

All those whose children were pre- or post-surgery had been informed that there was a chance of under- or over-correction.

*‘… he explained that it could make his eyes more or less aligned … it was an operation with obviously all the anaesthetic risks and everything but it wasn’t a major operation and they did hundreds every year … there were good results from having it done and that’s what he would recommend.’****4****–****12 group - surgery***

### Possible decision support

#### Clinicians

When asked how parents and children or they themselves could be better supported in making treatment decision, written information and a support network of other parents were mentioned. The idea of an information leaflet was not universally popular, however: some believed there were already too many information leaflets in clinics and X(T) was not a common enough condition to warrant one.

#### Parents

As with the clinicians, the two most commonly mentioned ways that parent/child decision-making could be supported were written information and the option to hear from parents whose child had undergone treatment. The majority of parents said that they struggled to retain what was conveyed during clinic visits, so it was not surprising that the provision of an information sheet was raised as a way to better support decision-making:

*‘Maybe getting something to take away to explain things a bit more clearly. But I know at the time when I’ve sat in the room with them and they’ve talked me through everything I’ve understood it all, and it’s made sense. I’ve been able to make my decisions from what they’ve given me erm but it’s when you walk out and a lot of that goes out of your head, so it would be nice to bring something back.’****Under 4 group - occlusion***

The opportunity to talk to other parents faced with the same decision was raised as a potential aid to decision making by a few parents, and explored with others. There were mixed views and the recognition that it may not suit the needs of all parents, but the majority thought it would be a good idea and there could be different ways to learn about the experience of others.

Another suggestion was that parents write down any questions they may have once they return home to take with them to the next clinic appointment.

## Discussion

In this study we wished to gain an understanding of how treatment decisions about X(T) are made, explore the factors and issues that shape these decisions, and try to identify ways to better support those involved. We have identified three key factors that could impact on treatment decision-making: information provision, parental engagement and clinician uncertainty.

### Information provision

Clear and reliable information is a key factor in good decision-making and issues around inadequate information were highlighted in this study. Despite reporting that clinicians provided sufficient information verbally, many parents in this study lacked understanding about the strabismus, treatment options and the rationale for treatment. Confusion may have been alleviated if clinicians had explained why certain treatment options were *not* worthwhile pursuing, for example treatments such as occlusion. A number of parents would have liked written information to reinforce the information given verbally and to share with other family members who often contribute to decision-making. The literature around the information needs of parents tends to focus on children with a life-threatening [20] or chronic illness [21], therefore it is difficult to draw comparisons with X(T) where the child is well and relatively symptom-free. There was one study, however, where the views of parents of children with more general healthcare needs were explored; they found that parents preferred verbal ‘one-to-one’ information but supplemented with written materials [22].

### Parental engagement

The findings reveal a varied picture around preferences for and actual decision-making for clinicians and parents. Slightly more clinicians expressed a preference for shared involvement in decisions than for the parent to make the final decision after seriously considering their opinion. However, the clinician interview data revealed that shared decision making was not widely practiced. A key feature of shared decision making is to incorporate patient preferences and values into treatment decisions. The factors and issues that shaped treatment decisions for parents were overwhelmingly appearance and a fear of their child being bullied and, for a few, around the impact of the strabismus on their child’s vision; it is the former that appears to be the main driver for treatment, primarily surgical intervention. In contrast, for clinicians, decisions for all treatments are based on clinical features. Parents did not always discuss their concerns about appearance and bullying when in consultations with the eye clinic team, although consultants and orthoptists were aware that this is often a worry for them. There appears to be a need for clinicians to spend more time exploring parents’ concerns and preferences, as well as their understanding of the condition and interventions available. For example, due to the intermittent nature of X(T), parents’ concerns about stigma and bullying at school are most likely unfounded and clinicians could have a role to play in explaining and providing reassurance around this issue. In some families there had been conflict between parents on the decision around surgical intervention and clinicians could have a role to play; exploring the understanding, values and preferences of both parents may have helped to facilitate decision-making and alleviate concerns.

For parents, slightly more expressed a preference to make the decision themselves - after considering the doctor’s opinion - than to share the decision with the clinician. The literature around parental decision-making preferences is limited. One study [21] of infants with atopic dermatitis reported slightly more parents expressed a preference to share the decision with the clinician than to make it themselves after considering the doctor’s opinion, yet a general finding was they felt their basic information needs around diagnosis and treatment had not been met. Their experiences of the care pathway were very different to those in our study, which makes it difficult to compare preferences around treatment decision-making.

### Uncertainty

Clinician uncertainty around the effectiveness of some treatments was an issue, particularly for some orthoptists, who felt this reflected negatively on their professionalism. None of the parents appear to have picked up on this, but clinician uncertainty may have had an impact on how they explained a treatment and its rationale. Some orthoptists attempted to keep abreast of X(T) research, for example through a journal club, but there remained a sense of frustration at the lack of, or contradictory, evidence on the success of treatments.

### Ways to improve decision-making

Potential ways forward to improve the decision making process for all concerned were identified: particularly written information for parents and learning about the experiences of other parents/children in the same situation. Nonetheless, some clinicians were critical of the idea of an information leaflet about X(T) and treatment, but this could be accessed via the hospital website if clinicians are concerned about yet another leaflet held in the clinic. On the same website videos or blogs from parents and children who have undergone the various management options could be accessed by interested parents.

Although not identified explicitly by clinicians, a method of assisting them to explain to parents the treatment options, together with the uncertainties, in a clear and concise way could be helpful. This extends the idea of written information into that of a concise brief decision aid, such as an option grid - a concise decision aid where answers to questions frequently asked by patients are provided for each treatment/management option for a particular health condition - which can support decision making both within and around the consultation [23]. This could be of particular benefit to orthoptists, who have the most regular contact with parents and children, and are more likely to suggest conservative treatments such as occlusion and minus lenses.

## Conclusions

This study has highlighted several areas where the experience of treatment decision-making could be improved for both clinicians and parents. Firstly, the provision of written information and opportunities for parents to learn about the experiences of others in the same situation, particularly if that also supports understanding of what is important to parents in decision-making. Secondly, more discussion with parents about their values and preferences would potentially help not only reveal differences between clinicians and parents in the factors that might influence decision-making, but also enable informed decisions to be jointly shared based upon both clinical factors and parents’ values. Finally, developing a formal decision aid to facilitate discussions between clinicians, parents and children on all treatment options would offer a solution that goes beyond straightforward information to support shared decision-making. The inclusion of current data on success rates would enable clinicians to convey with confidence the uncertainties around treatment and to discuss and engage parents’ concerns. Subsequent to, and informed by, this work, an option grid is being developed and has been used locally [24].

## Additional file

Additional file 1:
**Standards for Reporting Qualitative Research (SRQR).** (DOCX 14 kb)
